# Predictive factors of distal radioulnar joint instability after surgical treatment of distal radius fractures

**DOI:** 10.1097/MD.0000000000036505

**Published:** 2023-12-01

**Authors:** Chenfei Li, Lingde Kong, Xuyang Shi, Zuzhuo Zhang, Jian Lu, Bing Zhang

**Affiliations:** a The Third Hospital of Hebei Medical University, Shijiazhuang, Hebei, P.R. China.

**Keywords:** distal radioulnar joint instability, distal radius fracture, wrist

## Abstract

Distal radioulnar joint (DRUJ) instability is a common postoperative complication of distal radius fractures, seriously impacting patients’ quality of life. This study investigated its possible influencing factors to determine prognosis and to guide treatment better. We retrospectively included a series of patients with distal radius fractures that underwent volar locking plate fixation. Basic patient information and imaging parameters were collected. The incidence of DRUJ instability during follow-up was recorded, and factors associated with DRUJ instability were determined using univariate analysis and multifactorial logistic regression analysis. A total of 159 patients were enrolled in this study. At 6 months of follow-up, 54 patients (34.0%) had DRUJ instability, and multivariate analysis showed coronal plane displacement (OR, 1.665; 95% CI, 1.091–2.541), fracture classification (OR, 0.679; 95% CI, 0.468–0.984) and DRUJ interval (OR, 1.960; 95% CI, 1.276–3.010) were associated with DRUJ instability after volar locking plate. DRUJ interval, coronal plane displacement, and fracture classification are associated with DRUJ instability during follow-up. Therefore, preoperative risk communication and intraoperative attention to recovering relevant imaging parameters are necessary for these patients.

## 1. Introduction

Distal radius fractures (DRFs) account for approximately 1/6 of all fractures in the body and are the most common upper extremity fractures.^[1,2]^ For fractures of type B and C in the AO classification of DRFs, internal fixation with a volar locking plate (VLP) is the most common treatment modality to restore the flatness of their articular surfaces.^[3,4]^ Especially for young adults aged 18 to 65 years with high functional requirements of the wrist joint, surgical treatment can better achieve anatomical repositioning. The incidence of distal radial ulnar joint (DRUJ) instability after DRFs range from 2% to 37% and is a fundamental cause of ulnar wrist pain and can severely limit the rotation of the radius at the distal ulna.^[5]^

**Figure 1.**
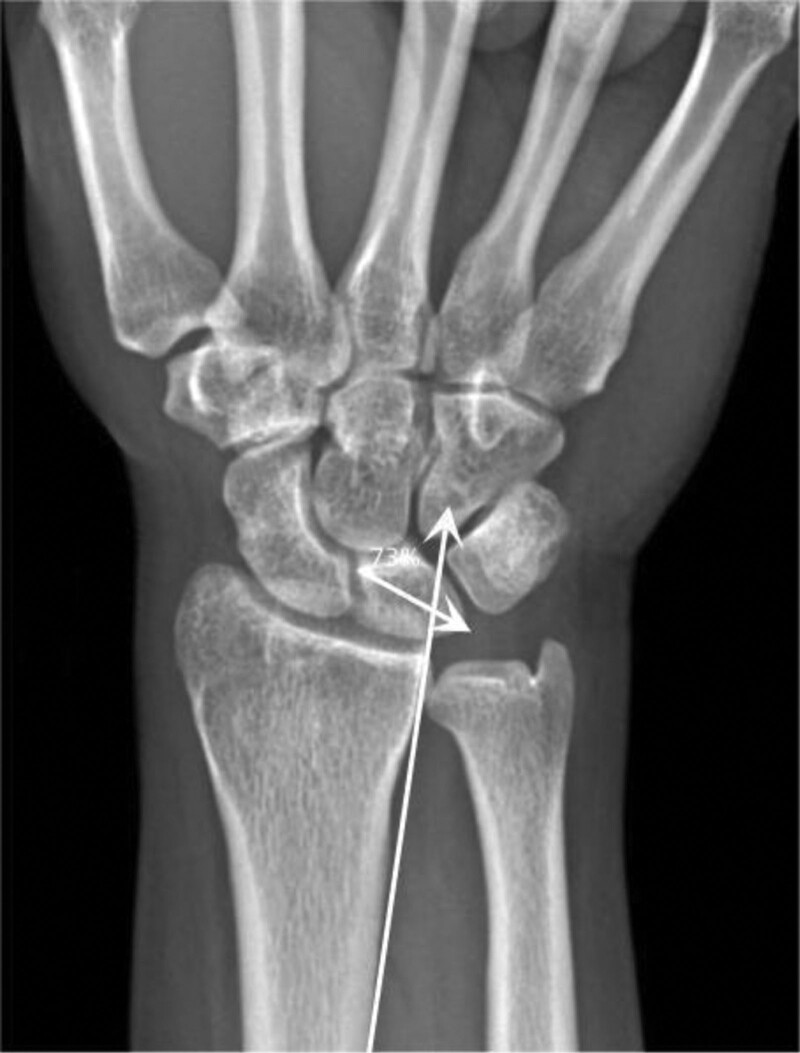
Measurement of radial translation.

Current research on DRUJ instability after DRFs is relatively limited, and its specific influencing factors are still controversial. Among the many possible influencing factors, triangular fibrocartilage complex (TFCC) injury can be considered almost necessary for DRUJ instability.^[6]^ Conservative treatment is still the first-line treatment for patients with TFCC tears.^[7]^ It is generally accepted that TFCC repair is not required in DRFs, and satisfactory DRUJ stability can be obtained by achieving anatomic repositioning of the bony structures.^[6,8]^ Coupled with TFCC injuries requiring magnetic resonance imaging for a definitive diagnosis, and magnetic resonance imaging is expensive and not routinely performed for DRFs, we need indicators to determine their prognosis in an X-ray accurately. Ross et al^[9]^ defined a “radial translation” in which a line is drawn on the X-ray in the ulnar direction from the proximal radius to the metaphysis and extends distally through the proximal carpus, and then a second line is marked on the X-ray along the lunate bone transverse width to assess the intersection point with the first line. In the above studies, the intersection issue had a mean of 45.48% (SD 14.19%; range 25%–73.68%) of the lunar remaining on the radial side.^[9]^ In our study, we measured the percentage of the lunate bone radial to that point (Fig. 1). This parameter reflects the displacement of the fracture in the coronal plane by describing the relative position of the radius to the lunate bone. However, there is still no definitive evidence-based proof of whether this coronal displacement is associated with the development of DRUJ instability after DRFs.

This study aims to identify the possible factors influencing DRUJ instability after DRFs, improve surgeons’ awareness of this complication, and more accurately determine prognosis and guide treatment.

## 2. Material and methods

### 2.1. Study design and patient population

We reviewed patients with DRFs who underwent VLP fixation at the Third Hospital of Hebei Medical University from January 2019 to March 2023. The inclusion criteria were 18 to 65 years old, AO type B or AO type C DRFs treated with VLP internal fixation. Exclusion criteria were open injuries, multiple fractures, previous DRFs, treatment other than VLP, combined neurovascular injuries, and incomplete data. The study was a retrospective observational study with a waiver of informed consent approved by the Ethics Committee of the Third Hospital of Hebei Medical University, and data were collected and analyzed using anonymous methods in all cases.

### 2.2. Data collection and parameter evaluation

This study analyzes the factors influencing postoperative DRUJ instability regarding patient-related factors, trauma-related characteristics, and preoperative X-ray parameters. Patient-related factors included age, gender, and other underlying diseases.

Trauma-related characteristics are collected from the patient electronic medical record, such as whether it is a high-energy injury or not and whether it is a dominant hand injury. Preoperative swelling assessment on the first day of hospitalization. If the wrist is swollen compared to the opposite side, but the skin texture can be identified, the swelling is considered mild. The node is considered severe if we cannot determine the skin texture or if blisters are present.

**Figure 2.**
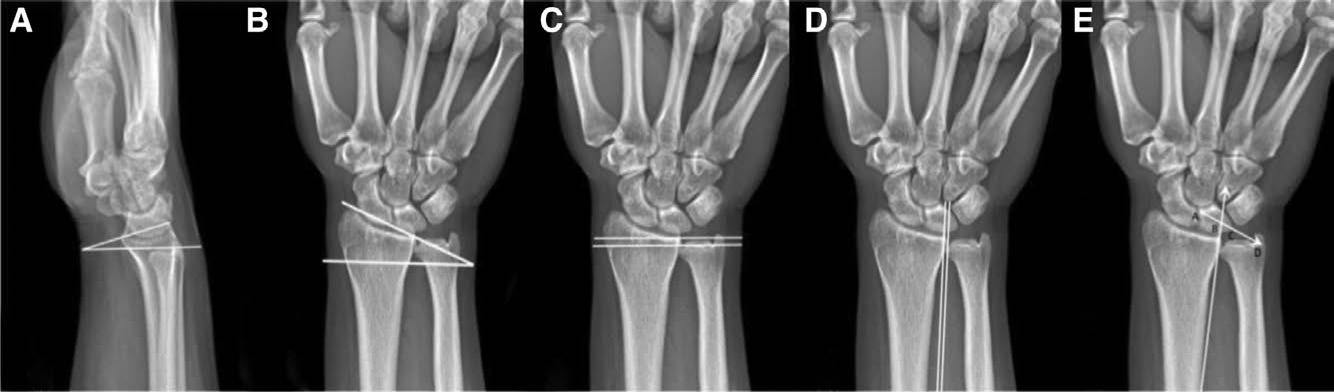
X-ray parameters measured in this study. (A) Volar tilt; (B) radial inclination; (C) ulnar variance; (D) DRUJ interval; (E) coronal plane displacement. DRUJ = distal radioulnar joint.

The experiment must measure 5 imaging parameters: volar tilt, radial inclination, ulnar variance, DRUJ interval, and coronal plane displacement (Fig. 2). The volar tilt refers to the angle between the line connecting the most distal point of the distal radial articular surface on the volar and dorsal sides and the vertical line of the long axis of the radius in a lateral view of the wrist. A radial inclination can be defined as a line connecting the most distal point of an ulnar radial side of a distal radius to the vertical line of the radius’ longitudinal axis. Do the vertical line of the radial midshaft through the ulnar lateral edge of the radial articular surface, and the minimum distance from the distal ulnar articular surface to this line is the ulnar variance. The ulna and radius are close to each other at the wrist joint, which constitutes the lower ulnar radial joint, and the gap between the ulna and radius is the DRUJ interval. Mark the radial and ulnar cortices of the lunar bone as points A and C, respectively, make an equal extension line CD of AC on the ulnar side, and record the intersection of the radial cortical extension line with AD as B. Record the ratio of BD to AB as the coronal plane displacement. Two blinded observers assess the radiological findings independently. For continuous variables, use the mean value. Disagreements between 2 observers on categorical variables were resolved by discussion, and if the conclusions could not be agreed upon, a third observer made the final decision. All observers had more than 10 years of clinical experience.

### 2.3. Treatment and follow-up

Under brachial plexus block anesthesia, place the wrist joint on the operating table. Upper arm inflatable tourniquet inflated to 250 mm Hg—incision between the radial carpal flexor and radial artery. Cut and separate the pronator quadratus from the distal radius. After identification of the fragment and traction exposure, it was first repositioned and fixed with K-wires. Direct visualization and intraoperative X-ray fluoroscopy confirmed it to be well moved. The volar plate was inserted into the fracture site and fixed with screws. The basal fracture of the ulnar styloid and the fractures with large displacement are generally fixed with K-wires, and the minor fractures are not treated. Then judge the stability of the DRUJ. In extreme pronation, the ulnar head is pushed dorsally and the distal radius is pulled palmar. Subsequently, in the extreme supination position, push the ulnar head palmar and the distal radius dorsally. For patients with DRUJ instability, K-wires were used to fix the ulna head and radius together. Intraoperative x-ray fluoroscopy showed good repositioning and then completed fixation. After saline irrigation, repair the pronator quadratus and suture and dress the wound long-arm cast or short-arm cast is then applied to immobilize. All procedures were performed by the same experienced surgeon.

Routine postoperative antibiotics are used for 24 hours to prevent infection. Patients usually perform activities such as flexion and extension of each joint on the second postoperative day. Follow up with the patients at 1, 3, and 6 months postoperatively. At each routine follow-up visit, radiographs were taken and DRUJ stability was evaluated. The patient was considered to have DRUJ instability when all 3 conditions were present: First, wrist pain limited to the lower ulnar radial joint or ulnar styloid process, aggravated by rotation and ulnar deviation; Second, elastic bulge compared with the healthy side, the ulnar tuberosity was seen to bulge dorsally or palmary, repositioned by pressure, and spring back to its original position by lifting the hand; tired, restricted movement, with rotation and ulnar deviation of the affected forearm <70% of that of the healthy side.

### 2.4. Statistical analysis

We performed all statistical analyses using the Statistical Package for Social Sciences (SPSS) 26.0 (IBM Corporation, Armonk, New York, USA). Quantitative data were tested using independent samples *t* test or Mann–Whitney *U* test, and qualitative variables were identified using Fisher exact probability method to identify differences between groups. Kappa and intraclass correlation coefficient statistics were used to evaluate the inter-observer reliability of qualitative and quantitative data in X-rays, respectively. After univariate analyses, variables found to be potentially associated with the DRUJ instability (*P* < .20) were included in the multivariate logistic regression models. *P* values <.05 were considered to be statistically significant.

## 3. Results

A total of 159 patients with DRFs who underwent VLP fixation were enrolled in this study. The mean age at surgery was 41.9 ± 6 years. There were 76 males (48.8%) and 83 females (52.2%). There were 62 ulnar styloid process fracture cases and 52 instances of sigmoid notch fracture. There were 78 cases (49.1%) of AO type B fractures and 81 cases (50.9%) of AO type C fractures. Of the 159 patients, 54 (34.0%) had postoperative DRUJ instability.

In the case group, 1 ulna fracture styloid process using the K-wires fixation. For the stability of DRUJ judgment in the process of operation, the case group patients appeared unstable DRUJ 1, there are 2 cases in the control group, were fixed with K-wires. All K-wires were removed at 4 to 9 weeks, with an average time of 6.5 weeks. One patient in both groups developed a superficial tissue infection. Reduction loss occurred in 2 patients in the DRUJ stable group but did not require surgical intervention. No hospital-based neurological symptoms, ulnar styloid nonunion, or other complications were observed in either group.

The patients’ basic information is presented in Table 1, and there was no significant difference between the 2 groups at baseline. The preoperative X-ray parameters of the patients were placed in Table 2, and a univariate analysis of the parameters of the 2 groups of patients was performed. We found that fracture classification (*P* = .044), fracture associated with the sigmoid notch (*P* = .153), preoperative ulnar variance (*P* = .161), preoperative coronal plane displacement (*P* = .039), and preoperative DRUJ interval (*P* = .005) were potential risk factors. There were no statistical differences in patients’ age, gender, BMI, smoking, alcohol abuse, osteoporosis, hypertension, diabetes, causes of injury, degree of swelling, whether dominant hand injury, time from injury to surgery, volar tilt, radial inclination, whether the cast was above the elbow, and whether the fracture involved the ulnar styloid process of the ulna. The Kappa and intraclass correlation coefficient values showed good to excellent inter-observer reliability for the X-ray parameter measurements (Tables 3 and 4).

**Table 1:** The comparison of basic data in patients with and without DRUJ instability.

Variables	Case group	Control group	*P* value
Age (yr)	42.2 ± 5.8	41.7 ± 6.1	.620
Gender			.867
Male	25	51	
Female	29	54	
BMI (kg/m^2^)	23.9 ± 4.2	24.2 ± 4.3	.675
Smoker			.824
Yes	10	17	
No	44	88	
Drinker			.592
Yes	19	32	
No	35	73	
Osteoporosis			.706
Yes	16	27	
No	38	78	
Hypertension			.469
Yes	19	30	
No	35	75	
Diabetics			.792
Yes	5	13	
No	49	92	
Cause of trauma			.392
High energy	36	62	
Low energy	18	43	
Preoperative swelling			.739
Slight	30	55	
Severe	24	50	
Dominant hand injury			.616
Yes	28	49	
No	26	56	
Time from injury to operation (d)	3.1 ± 2.5	3.3 ± 2.2	.605
Cast over the elbow joint			.856
Yes	17	31	
No	37	74	

BMI = body mass index, DRUJ = distal radioulnar joint.

**Table 2:** The comparison of radiographic data in patients with and without DRUJ instability.

Variables	Case group	Control group	*P* value
AO classification			.044
AO type B	20	58	
AO type C	34	47	
Associated ulnar styloid process fracture			.229
Yes	25	37	
No	29	68	
Associated sigmoid notch fracture			.153
Yes	22	30	
No	32	75	
Preoperative volar tilt (degree)	10.0 ± 3.7	10.1 ± 4.0	.879
Preoperative radial inclination (degree)	20.1 ± 6.4	20.5 ± 6.9	.723
Preoperative ulnar variance (mm)	2.1 ± 1.4	1.9 ± 1.0	.161
Preoperative coronal plane displacement	2.5 ± 2.2	3.2 ± 1.9	.039
DRUJ interval			.005
>2 mm	33	39	
<2 mm	21	66	

DRUJ = distal radioulnar joint.

**Table 3:** Inter-observer reliability of qualitative variables in X-ray parameters.

Parameter	Kappa value	*P* value
AO classification	0.886	<.001
Associated ulnar styloid process fracture	0.960	<.001
Associated sigmoid notch fracture	0.930	<.001
DRUJ interval	0.811	<.001

DRUJ = distal radioulnar joint.

**Table 4:** Inter-observer reliability of quantitative variables in X-ray parameters.

Parameter	ICC	*P* value	95% CI
Preoperative volar tilt (degree)	0.829	<.001	0.710–0.901
Preoperative radial inclination (degree)	0.798	<.001	0.671–0.880
Preoperative ulnar variance (mm)	0.880	<.001	0.798–0.930
Preoperative coronal plane displacement	0.906	<.001	0.835–0.947

CI = confidence interval, ICC = intraclass correlation coefficient.

In further logistic regression analysis (Table 5), coronal plane displacement (OR, 1.665; 95% CI, 1.091–2.541), fracture classification (OR, 0.679; 95% CI, 0.468–0.984), and DRUJ interval (OR, 1.960; 95% CI, 1.276–3.010) were associated with VLP internal fixation after the incidence of DRUJ instability was correlated. There is no significant correlation between DRUJ instability after VLP and whether the fracture involves the sigmoid notch (OR, 1.333; 95% CI, 0.925–1.921) or preoperative ulna variance (OR, 1.352; 95% CI, 0.889–2.056).

**Table 5:** Multivariate logistic regression analysis of factors associated with DRUJ instability.

	*P* value	Odds ratio	95% CI
Associated sigmoid notch fracture	.166	1.333	0.925–1.921
Preoperative ulnar variance (mm)	.193	1.352	0.889–2.056
Preoperative coronal plane displacement	.031	1.665	1.091–2.541
AO classification (AO B/AO C)	.033	0.679	0.468–0.984
DRUJ interval (>2 mm/<2 mm)	.004	1.960	1.276–3.010

CI = confidence interval, DRUJ = distal radioulnar joint.

## 4. Discussion

DRUJ instability is one of the common postoperative complications of DRFs, and its specific influencing factors have been controversial in previous studies. In this study, we reviewed only patients aged 18 to 65 years and undergoing VLP fixation, and the incidence of DRUJ instability was found to be 34.0% after 6 months of follow-up. DRUJ instability incidence was higher after VLP fixation in patients with coronal plane displacement, fracture type AO C, and distal radial ulnar joint interval > 2 mm at follow-up. Focusing on these factors can help surgeons identify high-risk patients and adjust their monitoring and follow-up decisions.

Previous studies have focused on 2 clinical trials to determine the stability of DRUJ. First is the pressure test proposed by Kleinman,^[10]^ in which the examinee palm is placed flat on the table, and the wrist is rotated anteriorly. The examiner applies pressure on the distal ulna to the palmar side, and the ulna moves palmarly and produces pain at the inferior ulnar radial joint. A positive pressure test is also a positive “piano key sign.” Secondly, Jupiter^[11]^ proposed the clunk test, in which the physician squeezes the distal radius and distal ulna with the thumb and index finger and then passively rotates the DRUJ forward. A positive impact test is defined as a pronounced clunk sound. However, there is still no gold standard for the diagnosis of DRUJ instability. In a retrospective study by van Leerdam et al^[12]^ performed compression and collision tests to detect DRUJ instability in 46 patients with DRFs, with a positive rate of 35% and 17%, respectively. The definition of distal radial, ulnar joint interval instability in this study not only focuses on the anatomical damage but also considers the degree of functional limitation of anterior and posterior rotation of the defective ulnar radial joint, which has a higher clinical significance than the compression test or the impact test alone.

The study on the anatomy and function of TFCC has been relatively sound, which consists of triangular fibrocartilage and ligamentous structures that support the DRUJ and ulnar carpal joint.^[13]^ Of the 8 ligaments that make up the TFCC, the palmar and dorsal ligaments of the radial-ulnar joint are critical to the stability of the DRUJ.^[14]^ They originate from the dorsal and palmar margins of the sigmoid notch and are divided into superficial and deep branches in the coronal plane. The deep attachment is attached to the central recess and confers high stability to the DRUJ. The distal radial ulnar joint interval > 2 mm usually indicates injury to the triangular cartilage, is the most closely related influencing factor to the distal radial ulnar joint after DRFs in this study, and can be used as an essential indicator to judge the prognosis.^[15]^

In the study by Ross et al,^[16]^ it was noted that the distal radial ulnar joint interval was strongly influenced by the body position at the time of the shooting, and there were many clinical cases with an average DRUJ interval but coronal plane displacement. This suggests that coronal plane displacement may have a higher sensitivity in determining whether DRUJ instability may occur after DRFs. In an earlier biomechanical study, coronal displacement was shown to seriously affect the dorsal stability of DRUJ.^[17]^ Soft tissue stabilizers of the DRUJ include, in addition to the TFCC, the pronator quadratus, the ulnar carpal extensor tendon, the DRUJ joint capsule, and the interosseous membrane, especially the distal oblique bundle of the interosseous membrane, which is present in most of the population.^[18]^ In a cadaver study by Moritomo et al,^[19]^ the distal interosseous membrane of the forearm acts as a secondary stabilizer of the DRUJ, and residual ulnar translational deformity of the proximal radius has the potential to cause tension of the distal interosseous membrane, which in turn affects the stability of the DRUJ. However, coronal displacement has not been included in statistical analysis in previous studies, and one of the importance of this study is to fill in this gap. In different angles and different research methods, it has been shown that coronal displacement can affect the stability of the DRUJ. This suggests that in future research and clinical work, we should improve our understanding and attention to coronal plane displacement, which will help us to adjust the treatment and follow-up strategy of patients.

In some previous studies, fracture involvement the styloid process of the ulna, fracture involvement the sigmoid notch, and ulnar positive variance was high risk factors for TFCC injury.^[20–23]^ However, these 3 factors were not statistically significant in this study. This may be due to the fact that fractures without displacement have less impact on the volar and dorsal radioulnar ligaments, or because the small sample size of this study reduced its statistical power. Compared to the above 3 influencing factors, the AO classification of fractures contains more information and is included in a large proportion of studies on DRFs. Boel et al reviewed 148 studies that explored factors influencing function after VLP, in which fracture type was the most frequently studied influencing factor.^[24]^ Fracture classification can not only reflect the damage degree of bone structure, but also indicate the risk of soft tissue injury. AO type C fracture often represents more tissue structure destruction, and higher surgical difficulty, which leads to worse prognosis.

There are still several limitations of our study. First of all, this study is a retrospective study, the small sample size affects the power of test, and future multicenter studies are still needed to validate its accuracy further. Secondly, the study has more stringent inclusion and exclusion criteria, which is a strength but also limits the application of the results. Considering that patients of different ages have different requirements for DRUJ function, elderly people were not included in this study. AO type A fractures were also not included in this study because they are mostly treated conservatively. These factors collectively lead to a relatively narrow applicability of the results, which will need to be supplemented by future research. Finally, the parameters included were relatively limited, whereas advanced images such as computed tomography may provide a more accurate and valid evaluation index.

## 5. Conclusions

In summary, at 6-month follow-up, 34.0% of patients with DRFs developed DRUJ instability after VLP internal fixation. During follow-up, DRUJ interval, coronal plane displacement, and fracture classification were factors associated with DRUJ instability. The relevant influencing factors should be brought to surgeons’ attention, especially coronal displacement, which has previously received less attention. Patients with high-risk factors were informed of the preoperative risks, and intraoperative attention was paid to restoring relevant imaging parameters.

## Author contributions

**Conceptualization:** Chenfei Li.

**Data curation:** Lingde Kong, Xuyang Shi, Zuzhuo Zhang.

**Project administration:** Bing Zhang.

**Supervision:** Jian Lu, Bing Zhang.

**Writing – original draft:** Chenfei Li.

**Writing – review & editing:** Chenfei Li, Lingde Kong, Xuyang Shi, Zuzhuo Zhang, Jian Lu, Bing Zhang.
